# Activation of FoxO1/SIRT1/RANKL/OPG pathway may underlie the therapeutic effects of resveratrol on aging-dependent male osteoporosis

**DOI:** 10.1186/s12891-020-03389-w

**Published:** 2020-06-12

**Authors:** Omnia Ameen, Rania I. Yassien, Yahya M. Naguib

**Affiliations:** 1 Clinical Physiology Department, Faculty of Medicine, Menoufia University, Menoufia, Egypt; 2 Histology and Cell Biology Department, Faculty of Medicine, Menoufia University, Menoufia, Egypt; 3 Physiology Department, College of Medicine and Medical Sciences, Arabian Gulf University, Manama, Bahrain

**Keywords:** Male osteoporosois, Type II osteoporosis, Aging, Resveratrol, FoxO1, SIRT1, RANKL, OPG

## Abstract

**Background:**

Age-dependent male osteoporosis remains a poorly studied medical problem despite its significance. It is estimated that at least 1 of 5 men will suffer from osteoporotic consequences. Given that multiple mechanisms are involved in the process of senescence, much attention has been given to compounds with polymodal actions. To challenge such a health problem, we tested here the therapeutic potential of resveratrol in male osteoporosis. We also studied the possible molecular mechanisms that may underlie resveratrol effects.

**Methods:**

Thirty male Wistar albino rats were used in the present study. Rats were divided (10/group) into: control (3–4 months old weighing 150–200 g receiving vehicle), aged (18–20 months old, weighing 350–400 g and receiving vehicle), and resveratrol treated aged (18–20 months old, weighing 350–400 g and receiving resveratrol 20 mg/kg/day for 6 weeks) groups. Assessment of serum calcium, phosphate, bone specific alkaline phosphatase, inflammatory cytokines, oxidative stress markers, and rat femur gene expression of FoxO1, SIRT1, RANKL and OPG proteins was carried out. Histopathological assessment of different levels of rat femur was also performed.

**Results:**

Age-dependent osteoporosis resulted in significant increase in serum levels of phosphate, bone specific alkaline phosphatase, hsCRP, IL-1β, IL-6, TNF-α, MDA, NO, and RANKL gene expression. However, there was significant decrease in serum level of GSH, and gene expression of FoxO1, SIRT1 and OPG. Osteoporotic changes were seen in femur epiphysis, metaphysis and diaphysis. Resveratrol restored significantly age-dependent osteoporotic changes.

**Conclusion:**

We concluded that resveratrol can play an important role in the prevention of male osteoporosis. Resveratrol can counter the molecular changes in male osteoporosis via anti-inflammatory, anti-oxidant and gene modifying effects.

## Background

Osteoporosis is a prevalent skeletal disease in elderly which is characterized by progressive decrease in bone mass and increase in risk of fractures [1]. Although osteoporosis represents a major health and societal burden for both men and women, only a minority of men are screened for osteoporosis or treated for fracture prevention [2]. Osteoporotic fractures represent a major public health problem worldwide because of the associated morbidity, mortality and costs. The financial burden of osteoporotic fractures includes both direct (hospital acute care, in-hospital rehabilitation, outpatient services, long term nursing care), as well as indirect (co-morbid conditions) costs which may constitutes up to 75% of the overall healthcare cost of osteoporotic fractures. Nevertheless, some costs remain difficult to quantify, such as the deterioration of the patient quality of life, or the time spent by the family members taking care of the patient [3, 4]. Traditionally considered as a disease of aging women, osteoporosis is becoming an increasingly important male health problem with one in three fragility fractures after the age of 50 years occurring in men [5]. Almost 30% of hip fractures occur in men, and mortality risk after a hip or femoral fractures is higher in men than women [6]. Greater frailty may partly explain the increased fracture-related morbidity and mortality in men [7].

Bone is a dynamic and highly active tissue that undergoes a remodelling process throughout life via the coupled action of bone-resorbing cells (osteoclasts) and bone-forming cells (osteoblasts) [8]. The main principle of bone remodelling is to restore microdamage, adapt the skeleton to mechanical loading and maintain calcium and phosphorus homeostasis [9]. Bone homeostasis is achieved by an extremely coordinated communication between osteoblasts and osteoclasts. Generally, there are two cytokines that are produced largely by bone marrow stromal cells and osteoblasts and are essential for osteoclast viability: macrophage colony-stimulating factor (M-CSF), and receptor activator of nuclear factor-kappa B ligand (RANKL). RANKL stimulate osteoclast differentiation and activation, and inhibit osteoclast apoptosis [10]. These processes are antagonized by osteoprotegerin (OPG), a natural decoy receptor of RANKL which is mainly secreted by stromal cells and osteoblasts [9, 10].

It is well accepted that there are two distinct types of osteoporosis: postmenopausal (type I), and senile (type II) osteoporosis. Type II senile osteoporosis was generally attributed to the aging processes such as osteoblast dysfunction [10]. Unlike postmenopausal osteoporosis which involves mainly trabecular bone and is manifested clinically by fractures of the distal radius and vertebrae, aging-dependent osteoporosis involves both trabecular and cortical bone with characteristic hip fractures in addition to vertebral fractures [11]. Aging is generally associated with a progressive pro-inflammatory status, a phenomenon referred to as “inflammaging”; there is an increasing body of evidence that pro-resorptive cytokines, such as interleukin (IL)-1, IL-6 and tumour necrosis factor-alpha (TNF-α) could be potentialy mediating age-dependent osteoporosis [12]. IL-1 production is increased in estrogen-deficient model systems [13]. In addition, the bone resorptive effects of TNF-α are well documented [14]. Several studies indicate that IL-6 plays a key role in mediating bone loss following estrogen deficiency [15]. Another key element in the skeletal deterioration associated with aging is the progressive free radical damage resulting from oxidative stress. The levels of reactive oxygen species (ROS) increase in bone with age and sex steroid deficiency [16]. The administration of antioxidants inhibits osteoblast and osteocyte apoptosis in the bone of ovariectomized or aged mice, denoting that oxidative stress may decrease osteoblast and osteocyte lifespan at the cellular level [16]. Oxidative stress may inhibit osteoblast formation. In fact, the attenuation of the Wnt signalling pathway that is critical for osteoblastogenesis by oxidative stress is mediated by the FoxOs transcription factors [17].

Several in vitro and in vivo studies have shown beneficial effects of resveratrol in osteoporosis. In vitro studies indicated that resveratrol was able to directly stimulate osteoblast proliferation and differentiation, inhibit formation and promoted its apoptosis of osteoclasts [18]. In vivo studies revealed that resveratrol was able to promote bone mineral density and inhibit bone loss in ovariectomized rats [19], in young rats under tail suspension [20], and in old rats under hind limb suspension [21]. Resveratrol has been shown to improve the bone mechanical tests in type II osteoporosis [8]. Nevertheless, the anti-osteoporotic effects of resveratrol have been poorly investigated in aging males. Accordingly, this study was designed to evaluate the therapeutic effects and the possible underlying mechanisms of resveratrol on type II osteoporosis in old male rats.

## Methods

All experiments were conducted in adherence to the Guiding Principles in the Use and Care of Animals published by the National Institutes of Health (NIH Publication No 85–23, Revised 1996). Animal care and use were approved by the Faculty of Medicine Menoufia University Ethics Committee.

### Animals

Thirty male Wistar rats were obtained from a local animal providing facility to be used in the present study. Rats were allowed to acclimatize for 10 days before the start experiments. Rats were given free access to normal chow diet and water in an air-conditioned room with a 12 h light-dark cycles. At the end of the study, rats were scarified by cervical dislocation.

### Experimental design

After acclimatization, rats were divided into the following groups (10 rats per group): control group (3–4 months old weighing 150–200 g), aged group (18–20 months old, weight 350–400 g), and resveratrol treated aged group (18–20 months old, weight 350–400 g). Rats in the resveratrol treated aged group received resveratrol (20 mg/kg/day for 6 weeks, Sigma-Aldrich Co., Mo, USA) via oral gavage, while those in the control and aged groups received equal amount of the vehicle (0.9% NaCl) via the same route.

### Blood sample collection

At the end of experiments after 6 weeks, rats were fasted overnight and anaesthetised by sodium thiopental (STP, 60 mg/kg intraperitoneal injection). Blood was collected via cardiac puncture. Blood samples were left for 30 min at room temperature to allow for proper coagulation. Blood samples were then centrifuged at 2000 rpm for 10 min and the serum was separated and collected. Serum samples were stored at − 20 °C for further investigations.

### Biochemical analysis

Serum levels of different biomarkers were detected by either quantitative sandwich enzyme immunoassay (ELISA) or colorimetric methods. Interleukin 6 (IL-6), interleukin 1β (IL-1β) and tumour necrosis factor alpha (TNF-α) (Quantikine® ELISA, R&D Systems Inc., MN, USA), nitric oxide (NO) (QuantiChrom™, BioAssay Systems, USA), high sensitivity C reactive protein (hsCRP) and bone specific alkaline phosphatase (BALP) (MyBioSource Inc., San Diego, CA, USA) were determined by ELISA technique using an automatic optical reader (SUNRISE Touchscreen, TECHAN, Salzburg, Austria). Glutathione (GSH) and malondialdehyde (MDA) (QuantiChrom™, BioAssay Systems, USA), calcium and phosphorus (ELITech, France) were determined by routine kinetic and fixed rate colorimetric methods on a Jenway Genova autoanalyser (UK).

### Analysis of gene expression quantitative RT-PCR (qRT-PCR)

To evaluate the effects of aging and or resveratrol treatment on the studied groups, we examined the mRNA expression levels of forkhead box protein O1 (FoxO1), sirtuin 1 (SIRT1), receptor activator of nuclear factor-kappa B ligand (RANKL), and osteoprotegerin (OPG) regulatory genes via real time quantitative reverse transcription-polymerase chain reaction (RT-PCR) assay as described previously [22–25]. Total RNA was extracted following grounding frozen femur bone specimens using TRI reagent (Sigma-Aldrich, UK). Reverse transcription of femur RNA was then performed using the high capacity RNA-to-cDNA kit (Applied Biosystems, CA, USA). Subsequently, the generated cDNA was used to measure mRNA expression for the selected genes. Gene specific primers were designed using Primer Express Software version 2.0 (Applied Biosystems, USA). Glyceraldehyde 3-phosphate dehydrogenase (GAPDH) was used as the housekeeping control gene (Table 1). RT- PCR assays were performed in duplicate for all target and housekeeping genes using Applied Biosystems 7500 FAST 96-well PCR machine (USA). With GAPDH serving as the endogenous control, relative mRNA expression of the gene of interest was calculated using the comparative Ct (2 − ΔCt) method. Data were expressed as a ratio (target gene/GAPDH), and were shown as the mean ± standard error of mean of at least three independent experiments.

**Table 1:** Primers used for measuring the expression of FoxO1, SIRT, RANKL and OPG genes

FoxO1	Forward	CACCTTGCTATTCGTTTGC
	Reverse	CTGTCCTGAAGTGTCTGC
SIRT1	Forward	AGA AACAATTCCTCCACCTGA
	Reverse	GCTTTGGTGGTTCTGAAAGG
RANKL	Forward	GACAGGCACGGACT CGTA
	Reverse	CGCTCATGCTAGTC GTCTA
OPG	Forward	TGGCACACAGTGATGAATGCG
	Reverse	GCTGGAAAGTTTGCTCTTGCG
GAPDH	Forward	TGCACCACCAACTGCTTAGC
	Reverse	GGCATGGACTGTGGTCATGAG

### Haematoxylin and eosin (H&E) stain

At the end of the experiment, the right femur of every rat was dissected and processed for histopathology examination. The excised parts were longitudinally cut at the level of the metaphysis, while they were cut transversely at the epiphysis and the diaphysis levels. Cut bones were fixed for 2 days in neutral buffered formaldehyde, then they were decalcified using the chelating agent ethylenediaminetetraacetic acid (EDTA) in its disodium salt form. The chelating solution was prepared from 5.5 g EDTA, 90 ml distilled water, and 10 ml formalin. The decalcification period was 4 weeks, and the solutions were changed daily. The decalcifying solution volume was always maintained approximately 30–50 times the volume of the tissue. Decalcified bones were then dehydrated in ascending grades of alcohol, cleared in xylene, and impregnated in paraplast for 3 h in an oven at 58 °C. Thereafter, decalcified bones were embedded in paraplast. At a thickness of 7 mm, serial sections were cut, stained with H&E and examined under a light microscope [26].

### Statistical analysis

Analysis of Variances (ANOVA) with Tukey’s post hoc tests were used for statistical analysis of the data using Origin® software. Results were expressed as mean ± standard error (SE), and *p* values < 0.05 were considered significant.

## Results

**Fig. 1:**
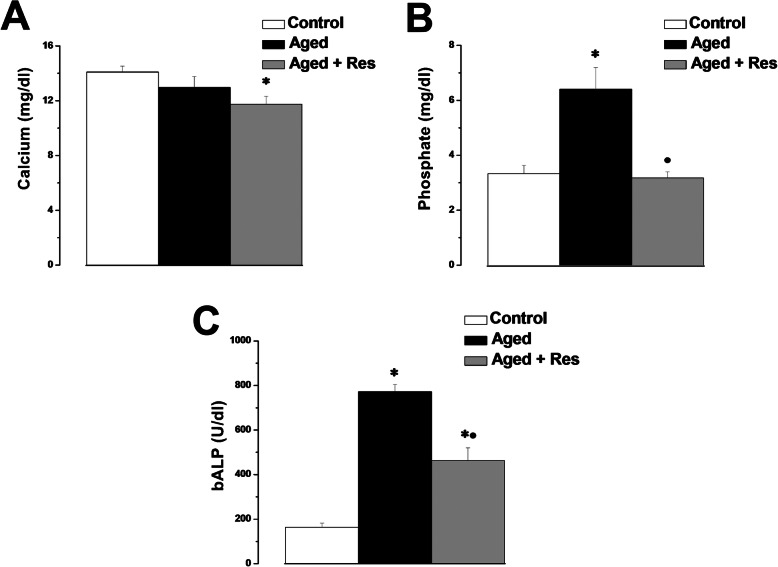
Serum calcium, phosphate and bone specific alkaline phosphatase levels amongst the studies groups. **a** Serum calcium levels in control (white column), aged (black column) and aged + resveratrol treated (grey column) groups. **b** Serum phosphate levels in control (white column), aged (black column) and aged + resveratrol treated (grey column) groups. **c** Serum bone specific alkaline phosphatase levels in control (white column), aged (black column) and aged + resveratrol treated (grey column) groups. (Significant = *p* < 0.05, * significant when compared to the control group, • significant when compared to the aged group. Number of rats = 10/group)

Serum calcium level showed insignificant difference between the aged and control rats (12.97 ± 0.8 vs 14.1 ± 0.44 mg/dl). However, serum calcium level was significantly lower in resveratrol treated aged group when compared to the control group (11.75 ± 0.59 mg/dl), while it remained insignificantly different when compared to the aged rats (Fig. 1a). Serum phosphate level was significantly higher in the aged group when compared to the control group (6.4 ± 0.79 vs 3.33 ± 0.29 mg/dl). Serum phosphate level was significantly lower in resveratrol treated aged group when compared to the aged group (3.18 ± 0.22 mg/dl), but was insignificantly different when compared to the control rats (Fig. 1b). Serum bone specific alkaline phosphatase was significantly higher in the aged group when compared to the control group (772.33 ± 32.68 vs 163.67 ± 18.59 U/dl). Serum bone specific alkaline phosphatase level was significantly lower in resveratrol treated aged group when compared to the aged group (463.1 ± 56.6 U/dl), but it was still significantly higher than the corresponding value in the control group (Fig. 1c).

**Fig. 2:**
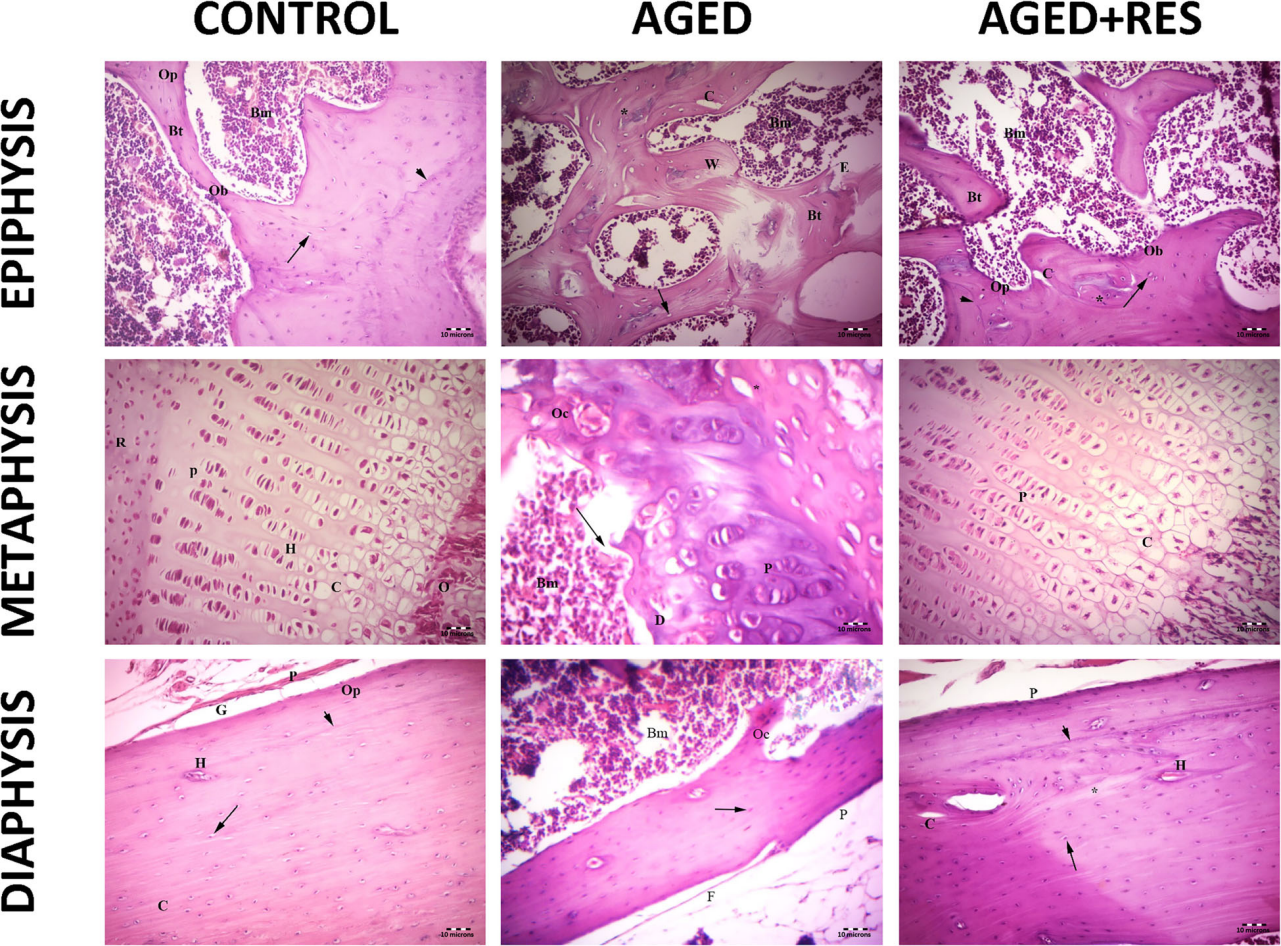
Histopathological photomicrographs of the studied groups. The upper left panel: the epiphysis of the control group showing the bone marrow spaces (Bm) between the irregular branching and anastomosing bone trabeculae (Bt) of cancellous bone. Osteoprogenitor cells (Op) and osteoblast (Ob) lining the endosteum with osteocytes inside the lacunae (↑) of bone trabeculae are seen. Cement lines are also seen (arrow head). Notice that the bone marrow is formed of hematopoietic tissue, scattered adipocytes, and blood cells. The upper middle panel: the epiphysis in aged group showing thin bone trabeculae (Bt) surrounding wide fatty bone marrow spaces (Bm). Refractile areas (*) appear inside the trabeculae. Osteoporotic cavities (C) and woven bone (W) in the trabeculae can be seen. Apparent decreased osteocytes (arrow) and eroded areas can also be seen (E). Notice that the bone marrow is fattier as compared with control. The upper right panel: the epiphysis in the resveratrol treated aged rats showing branching bone trabeculae (Bt) enclosing the bone marrow (Bm) spaces. The osteogenic cells (Op) lining the trabeculae are seen. Apparent increase in the number of osteoblasts (Ob) lining the endosteum of the bone trabeculae and the number of osteocytes (arrow) as compared to aged group. Cement line are present (arrow head). Small area of refractile bone (*) and few osteoporotic cavity (C) are still seen. The middle left panel: the metaphysis (epiphyseal plate) in control group showing the resting zone (R), the proliferating zone (P), the hypertrophic zone (H), and the calcified zone (C), followed by the zone of ossification(O). The regularly arranged cell columns with a basophilic matrix are detected. The middle central panel: the metaphysis in aged group showing irregularly arranged columns of cells in the proliferating zone (P) with degenerated cells (D). Wide bone marrow (Bm) and wide empty lacunae (*) are seen. Tear in bone (→) and osteoclasts (Oc) are seen. The middle right panel: the metaphysic in resveratrol treated group showing more regularity of both cells and columns in the proliferating (P) and calcification (C) zones compared with aged groups. The lower left panel: the diaphysis (shaft of femur) in control rat showing outer periosteum (P), Subperiosteal groove (G) and Haversian systems (H). Osteocytes in their lacunae (arrow), osteoprogenitor (Op) and regularly arranged collagen fibres (C) are seen. Cement lines (arrowhead) are detected. The lower middle panel: the diaphysis in aged rat showing eroded periosteum (P) and thinning of the outer fibrous layer(f) of the periosteum. An apparent decrease in the number of irregularly arranged osteocytes (↑) and fattier bone marrow (Bm) as compared with that of the control group . Notice that the shaft is apparently thinner than control. The lower left panel: the diaphysis in resveratrol treated aged rat showing outer periosteum (P) and many Haversion system (H). Nearly normal osteocytes in their lacunae (arrows) with an apparent increase in the number of osteocytes compared with that of the aged group and distinct cement line (arrow head) can be seen. Small osteoporotic cavities (C) and small area of osteolysis that appears as a palely stained area (*) are still detcted (H&E 200X)

**Fig. 3:**
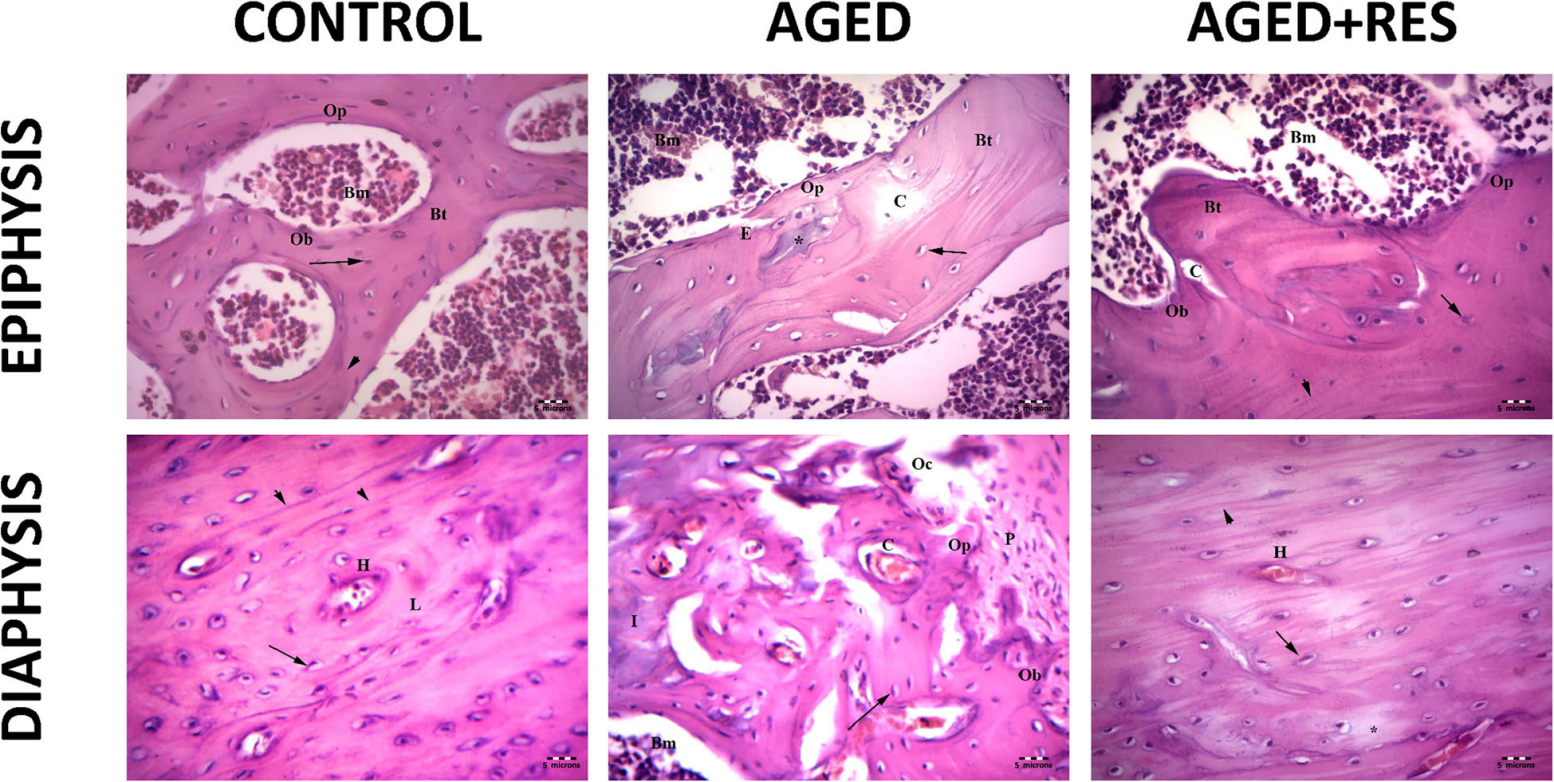
Anti-osteoporotic effect of resveratrol in aged rat femur in histological photomicrographs. Upper left panel: the epiphysis in the control group showing the Bone marrow spaces (Bm), irregular branching and anastomosing bone trabeculae (Bt). Osteoprogenitor cells (Op) and osteoblast (Ob), osteocytes (↑) and cement lines (arrow head) are also seen. Upper middle panel: the epiphysis in aged group showing thin bone trabeculae (Bt) surrounding wide fatty bone marrow spaces (Bm). Refractile areas (*), osteoporotic cavities (C), apparent decreased osteocytes (arrow) and eroded areas(E) can also be seen. Notice the few osteoprogenitor cells (Op) at endosteum. Upper right panel: the epiphysis in the resveratrol treated aged rats showing branching bone trabeculae (Bt) enclosing the bone marrow (Bm) spaces. The osteogenic cells (Op), apparent increase in the number of osteoblasts (Ob),the osteocytes (arrow) and cement line are present (arrow head). Small few osteoporotic cavities (C) can still be seen. Lower left panel: the diaphysis of a control rat, showing osteocytes (↑) around a centrally located Haversian canal (H). Cement lines (arrow head) and collagen fibres (L) are noticed. Lower middle panel: the shaft of a rat of aged group showing the eroded periosteum (P) with few osteoprogenitor (Op), osteoblast (Ob), multinucleated acidophilic osteoclast (Oc) in Howships lacunae and bone marrow (Bm. Less acidophilic bone matrix (I)) and multiple osteoporotic cavities containing osteoclast (C) can still be seen. Lower right panel: the shaft of a rat of resveratrol treated group showing many Haversion systems (H). Nearly normal osteocytes in their lacunae (arrows) with an apparent increase in their number as compared with that of the aged group and distinct cement line (arrow head) can be seen. Small area of osteolysis that appears as a palely stained area (*) is detected. (H&E 400X)

**Fig. 4:**
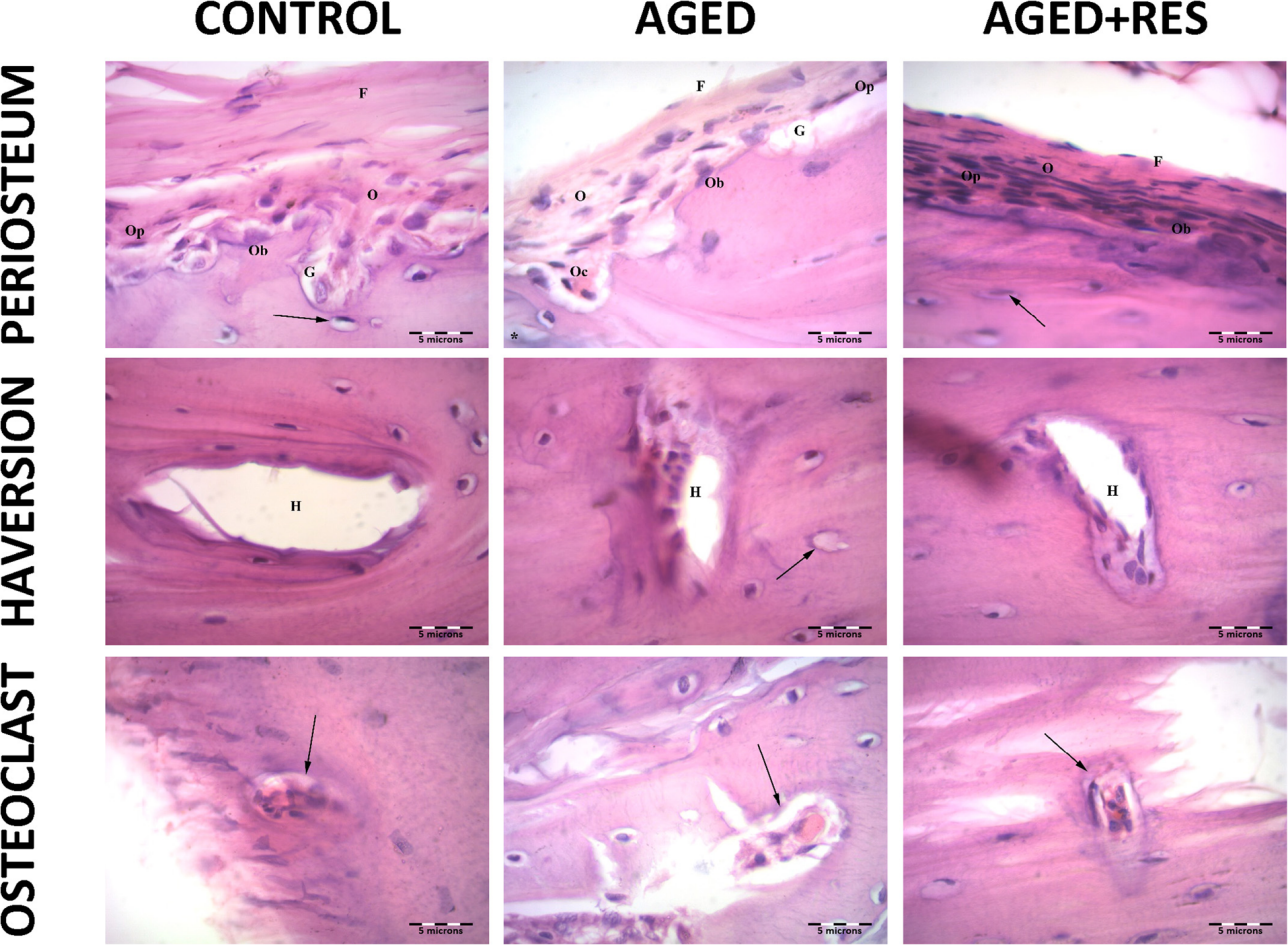
Photomicrographs showing resveratrol prevents aged-dependent histological changes in rat femur. Upper left panel: the shaft of femur of a control rat, showing the outer periosteum formed of an outer fibrous layer (f) and an inner osteogenic layer (O) which contains osteoprogenitor (Op) and osteoblast (Ob) cells and osteocytes in their lacunae (arrow). Subperiosteal grooves (G) are also seen. Upper middle panel: the shaft of aged rat, showing the outer periosteum formed of an outer fibrous layer (f) and an inner osteogenic layer (O) which contains osteoprogenitor (Op) and osteoblast (Ob) cells. Subperiosteal grooves (G) are also seen. Refractile area is seen (*). Notice that the periosteum appears thinner as compared to the control. Upper right panel: the shaft of the resveratrol treated aged rats showing the outer periosteum formed of an outer fibrous layer (f) and an inner osteogenic layer (O) which contain osteoprogenitor (Op) and osteoblast (Ob) cells and osteocytes in their lacunae (arrow). Notice that the periosteum appears thicker as compared to the aged, and nearly similar to the control rats. Middle left panel: the shaft of a control rat, showing wide Haversion system (H) surrounded by osteocytes in their lacunae. Middle central panel: the shaft of an aged rat, showing narrow Haversion system (H) surrounded by osteocytes in their lacunae and some lacunae are empty (arrow). Middle right panel: the shaft of a resveratrol treated aged rat, showing wider Haversion system (H) than aged and nearly similar to control surrounded by osteocytes in their lacunae. Lower left panel: the diaphysis of control rat showing multinucleated acidophilic osteoclast in Howships lacunae with ruffled border (arrow) lower middle panel: the shaft of an aged rat, showing multinucleated acidophilic osteoclast in Howships lacunae (arrow) surrounded by areas of erosion and osteolytic area of faintly stained bone. Lower right panel: the diaphysis in resveratrol treated aged rat, showing multinucleated acidophilic osteoclast in Howships lacunae with ruffled border (arrow). (H&E 1000X)

The histological examination showed sections from different levels of the rat femur stained with H&E. The epiphysis in the control group demonstrated normal histological appearance showing irregular cancellous bone with bone marrow spaces in between. Sections from the epiphysis in the aged group revealed loss of normal architecture of the cancellous bone with thin disconnected widely separated trabeculae. Some trabeculae showed refractile areas indicating bone loss and necrosis. Some eroded areas were seen on the bone surface. Moreover, there were numerous fat cells within the widening adjacent bone marrow. Sections from the epiphysis in the resveratrol treated aged rats showed apparently normal bone trabeculae, however, few areas of broken bone with erosion cavity could be seen in this group. Sections from the metaphysis of the control group demonstrated regularly arranged cell columns in characteristic zones: resting, proliferating, hypertrophic, and calcified zones, followed by the ossification zone. Sections from the metaphysis of the aged rats showed irregularly arranged columns of cells in the proliferating zone, with many degenerated cells and decreased basophilia of the matrix compared with the control group. In resveratrol treated aged group, the epiphyseal plate showed more regularity of cell columns in the proliferating zone with more basophilia of the matrix when compared to the aged non-treated rats. Sections from the diaphysis of the control group demonstrated the classical appearance of compact bone with many Haversian systems and covered by periosteum. Sections from the diaphysis of the aged group revealed that some osteocytes had wide lacunae, with an apparent decrease in the number of osteocytes when compared to the control group. Woven bone appeared with uneven staining of bone matrix with indistinct cement lines. Several resorption cavities were also seen within the matrix. Areas of palely stained osteoid matrix were noticed as well. Many multinucleated osteoclasts housed within erosion cavities could be seen. Sections from the diaphysis of the resveratrol treated aged rats showed marked improvement in bone structure when compared to the aged non-treated group. Nevertheless, some irregularly arranged osteocytes were seen within the bone matrix. However, the osteoclasts were fewer than those seen in the aged non-treated group (Figs. 2 and 3). The periosteum in the aged non-treated rats showed marked thinning, especially the fibrous layer, while it almost retained its normal thickness in the resveratrol treated group. Narrowing of Haversian canal was evident in the aged non-treated group, while it was almost normal in the resveratrol treated group. An area of faintly stained bone matrix with no osteocytes surrounded by osteoclasts in Howship’s lacunae was seen in the aged group. Small erosion cavities could still be seen in the resveratrol treated group (Fig. 4).

**Fig. 5:**
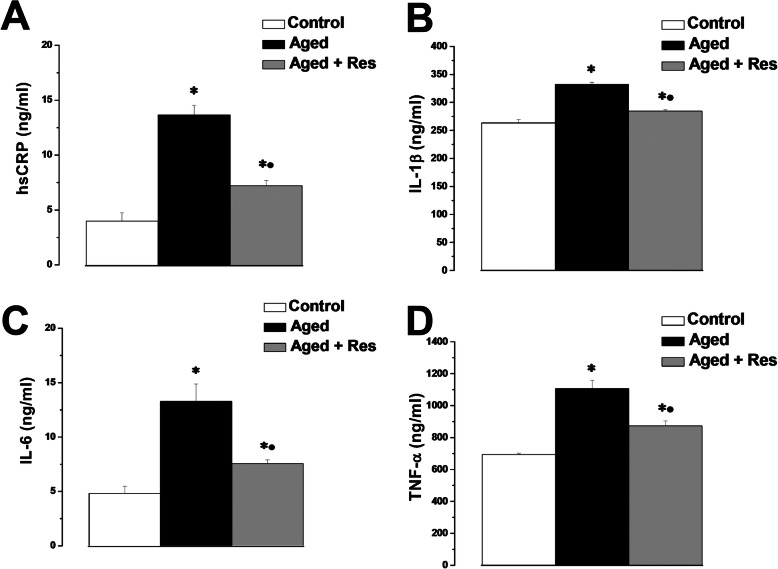
Resveratrol counters the altered inflammatory status in aged rats. **a** Serum high-sensitivity CRP levels in control (white column), aged (black column) and aged + resveratrol treated (grey column) groups. **b** Serum IL-1β levels in control (white column), aged (black column) and aged + resveratrol treated (grey column) groups. **c** Serum IL-6 levels in control (white column), aged (black column) and aged + resveratrol treated (grey column) groups. **d** Serum TNF-α levels in control (white column), aged (black column) and aged + resveratrol treated (grey column) groups. (Significant = *p* < 0.05, * significant when compared to the control group, **•** significant when compared to the aged group. Number of rats = 10/group)

The serum level of the pro-inflammatory biomarkers hsCRP, IL-1β, IL-6 and TNF-α were significantly higher in the aged rats (13.67 ± 0.88, 332.38 ± 3.8, 13.3 ± 1.58, 1106.18 ± 52.8 ng/ml respectively), when compared to the corresponding values in the control group (4.1 ± 0.76, 263.37 ± 6.78, 4.83 ± 0.66, 694.07 ± 7.12 ng/ml respectively). Treatment with resveratrol resulted in significant decrease in hsCRP, IL-1β, IL-6 and TNF-α levels (7.23 ± 0.47, 284.5 ± 2.19, 7.57 ± 0.35, 872.8 ± 32.29 ng/ml respectively) when compared to the aged group, however, their levels remained significantly higher when compared to the control group (Fig. 5a, b, c and d).

**Fig. 6:**
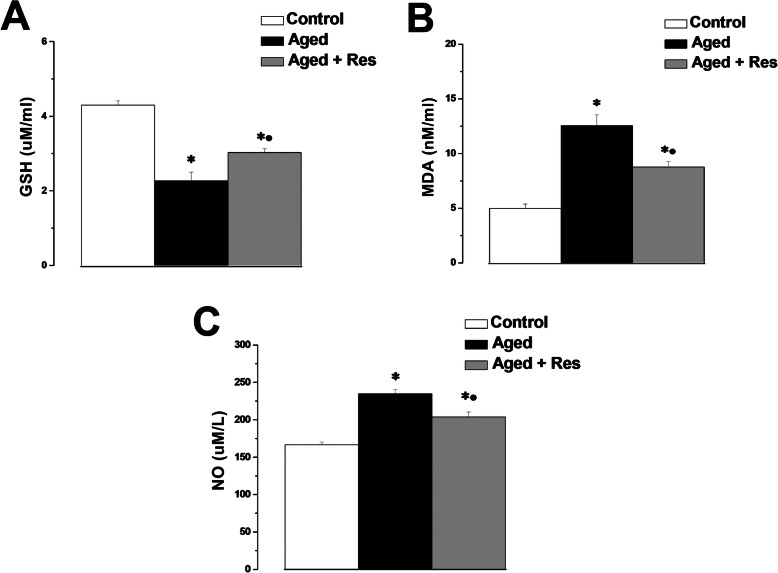
Resveratrol attenuates age-induced oxidative stress. **a** Serum GSH levels in control (white column), aged (black column) and aged + resveratrol treated (grey column) groups. **b** Serum MDA levels in control (white column), aged (black column) and aged + resveratrol treated (grey column) groups. **c** Serum NO levels in control (white column), aged (black column) and aged + resveratrol treated (grey column) groups. (Significant = *p* < 0.05, * significant when compared to the control group, **•** significant when compared to the aged group. Number of rats = 10/group)

There was significant decrease in the GSH level in the aged group when compared to the control group (2.27 ± 0.23 vs 4.3 ± 0.12 uM/ml). Serum GSH level was significantly higher in resveratrol treated aged group when compared to the aged group (3.03 ± 0.11 uM/ml), while it was still significantly lower when compared to the control rats (Fig. 6a). Expectedly, there was a significant increase in the MDA and NO levels in the aged group (12.57 ± 0.99 nM/ml and 234.78 ± 5.97 uM/l respectively), when compared to the corresponding values in the control group (5.1 ± 0.38 nM/ml and 166.67 ± 3.44 uM/l respectively). MDA and NO levels in the resveratrol treated group (8.78 ± 0.5 nM/ml and 204.07 ± 6.42 uM/l respectively) were significantly lower when compared to the aged group, however, they were significantly higher than the corresponding values in the control group (Fig. 6b and c).

**Fig. 7:**
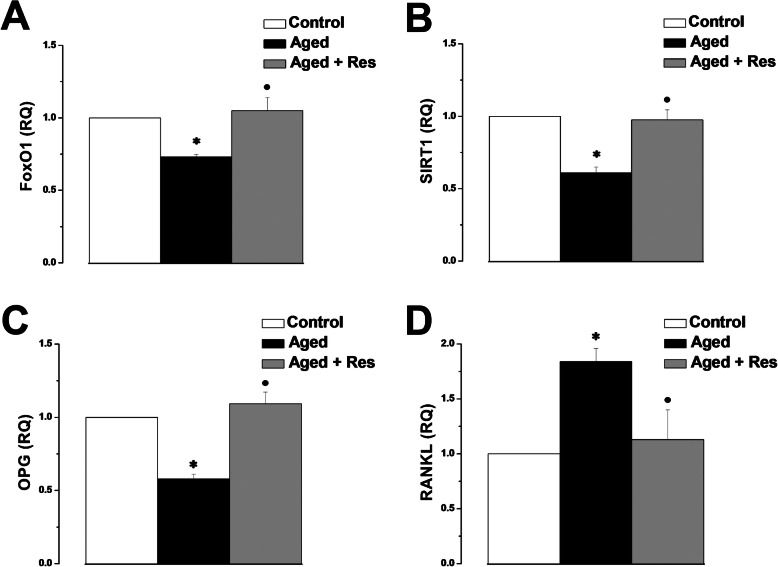
Effect of resveratrol on gene expression. **a** FoxO1 gene expression in control (white column), aged (black column) and aged + resveratrol treated (grey column) groups. **b** SIRT gene expression in control (white column), aged (black column) and aged + resveratrol treated (grey column) groups. (Significant = *p* < 0.05, * significant when compared to the control group, **•** significant when compared to the aged group. Number of rats = 10/group)

Expression of the FoxO1, SIRT1 and OPG genes (0.73 ± 0.02, 0.61 ± 0.04 and 0.58 ± 0.03 respectively), was significantly lower in the aged rats when compared to the control group (1). FoxO1, SIRT1 and OPG gene expression was significantly higher in the resveratrol treated rats (1.05 ± 0.09, 0.98 ± 0.07 and 1.09 ± 0.08 respectively), when compared to the aged group. RANKL gene expression was significantly up-regulated in the aged group when compared to the control group (1.84 ± 0.12 vs 1). RANKL gene expression was significantly lower in the resveratrol treated rats (1.13 ± 0.27), when compared to the aged group. There was insignificant difference in FoxO1, SIRT1, OPG and RANKL gene expression between resveratrol treated aged group and the control group (Fig. 7).

## Discussion

Aging is a progressive decline of the natural homeostatic mechanisms, leading to deterioration of tissues and organs functions with subsequent deleterious health outcomes [8]. Osteoporosis is a skeletal disorder characterized by low bone mass, structural weakening, decreased bone strength and increased risk of fractures resulting in rapid growth osteoporosis related morbidity amongst the elderly [27]. Osteoporosis represents a major health and societal burden in men as well as in women, nevertheless, not often men are screened for osteoporosis [2]. Consequently, finding new therapeutic approaches to slow down age-related osteoporosis has been a target for researchers. Rat model of male osteoporosis help in better understanding of the disease, and therefore, test for possible therapies. It has been reported that 9 months old male rats started showing age-related bone loss [28]. Rats used in the present study are comparable to 55–60 years old male humans [29]. Resveratrol is a polyphenolic compound naturally present in grapes, cranberries, and nuts. There is a growing body of evidence that resveratrol may be an effective therapeutic agent for age-related degenerative diseases including osteoporosis [30]. Resveratrol is able to target cytomembranes, intracellular receptors, signalling molecules, enzymes, oxidative system, DNA repair system, and transcription factors [31]. We demonstrated here a potential therapeutic role of resveratrol on male osteoporosis. We also elucidated that resveratrol anti-osteoporotic effects may involve the employment of FoxO1/SIRT1/RANKL/OPG pathway.

Serum BALP, phosphate and calcium are classical bone turnover markers. In the present study the mean values of serum bone specific alkaline phosphatase and phosphate were significantly higher in the aged group when compared with the control group. Similar results were reported previously [32, 33]. BALP is an important enzyme for osteoid formation and mineralization, and can be used as an index for the rate of overall bone turnover presenting the relation between bone resorption, bone formation and bone mineralization; the high bone turnover rate in osteoporosis is associated with increased serum BALP [32]. Another important indicator of the rate of bone remodelling is the concentration of serum phosphate. Disproportionate increase in bone resorption will lead to a higher plasma phosphate concentration, whereas increased bone mineralization causes lower serum phosphate level [34]. Resveratrol treated aged rats had significantly lower serum BALP and phosphate values when compared to aged non-treated rats. Evidence has shown that resveratrol has the capability of inhibiting osteoclasts differentiation, activity and accordingly bone turnover [18]. The inhibitory effect of resveratrol on osteoclast differentiation was associated with decreased serum BALP [35]. The mean value of serum calcium was insignificantly different in the aged group when compared to the corresponding value in the control group. This result was in agreement with previously published reports [32]. Nevertheless, serum calcium was suggested to be decreased in postmenopausal women with osteoporosis [33]. In our hands, administration of resveratrol in aged male rats led to decrement in serum calcium level reflecting a possible role of resveratrol in enhancing bone calcium deposition. Resveratrol has been shown to stimulate osteoblast activity, and therefore, increase bone mineralization [36]. In support to our results, it was reported that a transient decrease in serum calcium typically occurred within the first few weeks after administration of a potent anti-resorptive agents [37].

In the present work, histopathological findings demonstrated clearly the age-dependent osteoporotic changes in male rat femurs. Changes included significant decrease in the thickness of cortical and cancellous bone, widely separated bone trabeculae, osteoporotic cavities, irregularly eroded endosteal surfaces and woven bone in the trabeculae, and apparent decrease in number of osteocytes. Bone loss in osteoporosis could be initiated by the increase in depth of erosion cavities causing disruption of the trabeculae and perforation, leading eventually to conversion of the trabecular plates to widely separated rods and bars [38, 39]. The formation of erosion cavities with active brush border may result from the increase in the number of active osteoclasts, with subsequent bone resorption and rarefaction [40]. A possible relationship between abnormal lipid metabolism and osteonecrosis may coexist based on the presence of excess fat cells in the bone marrow of the aged rats [41]. Resveratrol substantially improved age-dependent histopathological changes in aged rats. Indeed resveratrol was reported to improve the microstructure of aged male rat femurs [8].

In the present study the serum level of the pro-inflammatory markers hsCRP, IL-1β, IL-6 and TNF-α was significantly higher in the aged group when compared to the corresponding values in the control group. Aging is associated with chronic low-grade increase in the circulating levels of inflammatory markers, which may be associated with low bone density [42, 43]. During the aging process, tissues release cytokines such as IL-1β, IL-6, and TNF-α, proteins such CRP, and pro-inflammatory transcription factors such as the nuclear factor kappa B (NFκB). The circulating inflammatory mediators have been implicated in the pathogenesis and progression of tissue alteration and failure in the elderly [24]. Pro-inflammatory cytokines might induce the formation of reactive oxygen species (ROS) which could trigger an inflammatory response through the activation of transcription factor NFκB [44]. NF-κB then translocates into the nucleus where it activates a variety of inflammatory genes such as inducible nitric oxide synthase (iNOS), COX-2, IL-1β, IL-6, IL-8, TNF-α and monocyte chemoattractant protein- 1[45]. Consecutively, IL-1β and TNF-α could activate NF-κB forming an amplifying feed-forward loop; a vicious cycle that may eventually lead to cell, tissue or organ dysfunction [8, 42]. Resveratrol has well proven anti-inflammatory activities. Resveratrol supplementation can directly suppress the release of the proinflammatory cytokines TNF-α, IL-1β, IL-6, IL-10, monocyte chemoattractant protein-1 (MCP-1), interferon alpha (IFN-α), and IFN-β in a wide range of rodents tissues [18, 46].

The serum levels of MDA and NO in the present investigations were significantly higher, while GSH was significantly lower in the aged group when compared to the corresponding values in the control group. The administration of resveratrol significantly restored the redox balance. Age-dependent oxidative stress occurs when the production of ROS exceeds the antioxidant defence capacity [24]. ROS promotes lipid peroxidation, oxidation of proteins and nucleic acids, and structural alteration of the membranes resulting in cellular damage [47]. Oxidative stress alters bone remodelling process causing an imbalance between osteoclast and osteoblast activities. This can lead to metabolic bone diseases and contribute to the pathogenesis of osteoporosis [17]. Age-dependent bone loss differs from estrogen-dependent osteoporosis in the progressive loss of osteoblasts activity rather than the enhanced osteoclast activity. The reduction in osteoblast activity during aging could be caused by an accumulation of adipocytes at the expense of osteoblasts in the bone marrow. Increased adipocytes in bone marrow may result in oxidative stress due to higher susceptibility to lipid peroxidation [48]. Oxidative stress stimulates osteoclastogenesis; significant increase in the number and activity of osteoclasts was observed when H_2_O_2_ was added to the cultures of human bone marrow mononuclear cells [44]. Furthermore, ROS induce the apoptosis of osteoblasts and osteocytes, thus favouring osteoclastogenesis [49]. Excessive apoptosis of osteocytes is correlated to an increased oxidative status causing an imbalance in favour of osteoclastogenesis [50]. Several factors produced by osteoblasts and osteocytes, most importantly the ligand of receptor activator of NFkB (RANKL) and osteoprotegerin (OPG), regulate both osteoclasts and osteoblasts activities. RANKL is produced by osteoblasts and activates the differentiation and activity of osteoclasts by interacting with specific receptors in preostoeclasts and mediates osteoclastogenesis and bone resorption [47]. Also, RANKL promotes the accumulation of H_2_O_2_ in osteoclasts and in their progenitors, which in turn improves osteoclasts proliferation. Therefore, increased ROS level, particularly H_2_O_2_, is a critical regulatory step in osteoclastogenesis and bone resorption [51]. OPG, a soluble receptor capable of binding and blocking RANKL, is produced by the activation of the signalling pathway Wnt/βcatenin resulting in inhibition of osteoclasts activity. Oxidative stress blocks the activation of osteoblasts and thus the production of OPG; enabling the action of RANKL to prevail with subsequent promotion of osteoclast differentiation and activity. The increase in RANKL/OPG ratio is, in fact, an index for the intensity of bone resorption [47]. Amongst all herbal medicines, recently termed natureceuticals, resveratrol health benefits have been well documented. Most of resveratrol therapeutic effects are owed to its antioxidant properties. It was reported earlier that resveratrol can act as a scavenger of superoxide and hydroxyl radicals, and peroxynitrite. Resveratrol was also reported to be capable of activating several antioxidant enzymes [52]. We could assume that the improvement in osteoporotic changes in our work could be partly due to the antioxidant properties of resveratrol.

It was important then to identify the possible molecular mechanisms that may underlie resveratrol effects on age-dependent osteoporosis in aged males. The real-time PCR results for the FoxO1, SIRT1 and OPG genes demonstrated a significant down-regulation of their gene expression in aged rats, while there was a significant up-regulation of RANKL gene expression. Resveratrol treated rats showed up-regulation of the FoxO1, SIRT1 and OPG gene expression, with concomitant down-regulation of RANKL.

SIRT1 is the first member of the Sirtuin protein family to be discovered. SIRT1is a longevity associated protein; activation of SIRT1 in mice was associated with a delay in the onset of many aging-related diseases, including osteoporosis [53]. Enzymes associated with SIRT1 are histone acetylation enzymes and, therefore, can regulate several molecules including NF-κB, enabling SIRT1 to regulate inflammation [54]. It has been reported that resveratrol-mediated SIRT1 activation can inhibit the NF-kB signalling pathway promoting osteoblasts differentiation [35, 39, 48]. Additionally, resveratrol can elicit a SIRT1-dependent inhibition of osteoclastogenesis [55]. Human adult retinal pigment epithelial (RPE) cells pre-treated with the SIRT1 activator SRT1720 showed abrogation of IL-8, IL-6 and MMP-9 expression [56]. The anti-apoptotic and anti-oxidant effects of resveratrol were abolished by SIRT1 knockdown in C2C12 myoblast cells; suggesting that SIRT1 is pivotal in mediating resveratrol-induced cell protecting effects [57]. SIRT1 siRNA blocked the anti-osteoporotic effect of resveratrol in ovariectomized rat model strengthening the assumption that resveratrol exerts its anti-osteoporotic action via SIRT1-NF-κB pathway [27, 35]. FoxO1, a member of the Forkhead box O family of proteins, is the most abundant isoform in osteoblasts. Accordingly, FoxO1 is thought to control bone formation through osteoblasts proliferation and differentiation, and redox balance [58]. FoxO1 can counteract the generation of ROS by over-expression of the antioxidant enzymes such as glutathione peroxidise and superoxide dismutase [59]. It has been reported that in hematopoietic stem cells FoxO1 reduces ROS by up-regulating the expression of anti-oxidant enzymes, whereas FoxO1 deletion led to an increase in osteoclast progenitors in the bone marrow [60]. FoxO1 is a target for SIRT1; SIRT1 appears to shift the FoxO1-dependent response towards the antioxidant activity and redox balance [57]. Receptor activator of nuclear factor-κB (RANK) is a member of the tumor necrosis factor family expressed by osteoclasts. The final common pathway in the regulation of bone resorption involves the interaction of RANK with its ligand (RANKL) [61]. Inhibiting RANKL significantly affects bone metabolism, and therefore, is a reasonable therapeutic strategy for the treatment of osteoporosis and other bone diseases characterized by increased bone turnover. OPG is the natural inhibitor of RANKL; Osteoporosis developed in OPG-deficient mice, while over-expression of OPG in mice inhibited osteoclastogenesis and improveded bone mass [62, 63]. Taken together, resveratrol seems to be able to shift the RANKL/OPG pathways toward osteobalstogenesis in age-dependent male osteoporosis.

## Conclusion

The present study demonstrated that treatment with resveratrol could guard against age-dependent osteoporosis in males both on the functional and structural levels. By means of its versatile actions, resveratrol ameliorated the inflammatory and oxidative stress conditions commonly present with senescence, and therefore averted the age-induced deleterious effects on the bone. The anti-osteoporotic effect of resveratrol could be mediated, at least in part, by altering the FoxO1/SIRT1/RANKL/OPG pathway. We report here a novel effect and underlying mechanism of resveratrol on type II osteoporosis.

## Data Availability

Data supporting findings are presented within the manuscript.

## Abbreviations

BALP: Bone specific alkaline phosphatase
FoxO1: Forkhead box protein O1
GSH: Glutathione
hCRP: high sensitivity C reactive protein
IL: Interleukin
M-CSF: Macrophage colony-stimulating factor
MDA: Malondialdehyde
NFκB: Nuclear factor kappa B
NO: Nitric oxide
OPG: Osteoprotegerin
ROS: Reactive oxygen species
RANKL: Receptor activator of nuclear factor-kappa B ligand
SIRT1: Sirtuin 1
TNF-α: Tumour necrosis factor-alpha
